# Saving Lives in Health: Global Estimates and Country Measurement

**DOI:** 10.1371/journal.pmed.1001523

**Published:** 2013-10-01

**Authors:** Daniel Low-Beer, Ryuichi Komatsu, Osamu Kunii

**Affiliations:** The Global Fund to Fight AIDS, Tuberculosis and Malaria, Geneva, Switzerland

## Abstract

Daniel Low-Beer and colleagues provide a response from The Global Fund on the *PLOS Medicine* article by David McCoy and colleagues critiquing their lives saved assessment models.

*Please see later in the article for the Editors' Summary*

**Linked Policy Forum**

This Perspective discusses the following new study published in *PLOS Medicine*:

McCoy D, Jensen N, Kranzer K, Ferrand RA, Korenromp EL (2013) Methodological and Policy Limitations of Quantifying the Saving of Lives: A Case Study of the Global Fund's Approach. PLoS Med 10(9): e1001522. doi: 10.1371/journal.pmed.1001522

David McCoy and colleagues critique the dominance of “lives saved” models of assessing the impact of health programs, using The Global Fund as a case study.

One of the most compelling reasons for development aid to health is that it saves lives, often for a few hundred dollars per year of life saved. Relatively uniquely in development, health has a set of high-impact interventions that can save lives directly. Insecticide-treated bednets (ITNs) protect families from malaria, antiretrovirals (ARVs) reduce mortality from HIV, and tuberculosis detection and treatment reduce TB mortality. Prevention activities, particularly for HIV, can save millions more lives. Yet, health programs have not always communicated with simple methods the lives they save.

In this week's *PLOS Medicine* David McCoy and colleagues discuss the “lives saved” model of The Global Fund to Fight AIDS, Tuberculosis and Malaria (The Global Fund). The Global Fund, together with WHO, UNAIDS, and scientists from the article by McCoy and colleagues [1],[2], have published simple peer-reviewed methods to calculate the lives saved from a restricted set of HIV, TB, and malaria interventions that have known mortality outcomes [3]–[7]. Our method includes only those health interventions with known, documented mortality effects: ARV treatment; directly observed treatment, short-course (DOTS); and ITNs. Our methodology uses documented data reported to the Global Fund on the individuals receiving these services. These results are first verified by national disease programs (we invest 5%–10% of our funds to build the capacity of country monitoring and evaluation systems), then by the Global Fund (which uses independent local fund agents to check the national data systems measuring these services every six months), and finally by on-site checks in a sample of health facilities to verify that people receive these services (as part of performance-based funding) [8].

In addition, the Global Fund's method applies the agreed, partner mortality estimates and models from WHO and UNAIDS [4] to these service results—for example, the latest scientific data on how HIV treatment or TB treatment will reduce the chance that a person will die of HIV or TB.

Extensive criteria are used to exclude countries where The Global Fund is not a significant contributor; that is, where The Global Fund does not contribute at least US$50 million; is a significant percentage of HIV, TB, and malaria spending; and does not support a key national-level activity, such as drug procurement. Where this does not occur, as has been the case in Uganda, Kenya, or South Africa in recent years, the results are not included.

The method to assess lives saved provides a conservative estimate. The estimate [3],[4] does not include the impact of HIV prevention (which in certain countries—e.g., Thailand, Uganda, Kenya, and Zimbabwe—has saved several million lives per country); the impact of malaria outside Africa and among adults; and the significant, secondary impact of DOTS treatment on reducing TB (as shown by the declines in TB prevalence in China, and in TB prevalence by 45% in Cambodia). Furthermore, reporting of services by programs in country are subject to substantial delays before they are reported globally. The most recent scale up in ITNs and ARV treatment are not fully included; for example, the lives saved are only half the number of people reported on ARVs. We do acknowledge the method [3],[4] has major limitations. Most importantly, it does not directly measure mortality, because in many countries in which we work vital registration systems are too weak, so the method is based on the latest partner estimates of mortality from WHO and UNAIDS.

The article in this week's *PLOS Medicine* by David McCoy and colleagues has great value in discussing the assumptions in the methods the Global Fund uses to assess lives saved and the partner estimates—of ARV adherence, use of ITNs, and the limitations of focusing only on a limited set of services. We agree that assumptions require additional sensitivity analysis, and we will update our estimates in 2014 as modeling is refined with new and improved data from country impact evaluations and updated WHO and UNAIDS estimates. We have published more detailed analysis of the ARV, ITN, and DOTS estimates as used by the McCoy and colleagues [4]. Yet, the uncertainty ranges, with the lives saved from ITNs as low as 27,000, were based on very limited data and provided little additional value. We fully agree with the need for increased country data on estimates and mortality assumptions of lives saved. Most importantly, global modeling needs strengthening with wider and deeper country measurement of epidemic trends and lives saved.

To significantly strengthen our assessment of lives saved, The Global Fund approved a new evaluation plan in 2012, which includes country health, HIV, TB, and malaria reviews to more directly measure mortality and impact on HIV, TB, and malaria trends in 25 countries, where 65% of the global burden of these diseases occurs [9]. To strengthen these direct country data and to put global commitments made with WHO and other partners into practice, we are also investing in five components of data systems: surveys, health information systems, vital registration, financial tracking, and country analytical capacity [10]. These investments in country data and analysis will form a basis to implement some of the recommendations to global modeling provided by McCoy and colleagues [1]. Our initial country reviews provide direct evidence of impact. For example, in Cambodia, malaria deaths have been reduced by over 80%, child mortality has declined in Tanzania, TB has declined in China, HIV prevalence has declined in Zimbabwe (though not in other countries, such as Uganda in recent years) [7],[11]. Some of these declines are not captured by global estimates of lives saved, and suggest these estimates may be conservative and require updating as country-level analysis of mortality is available.

The need for improved country data does highlight some of the weaknesses we see in the paper by David McCoy and colleagues [1]. Their analysis provides very little additional country data on uncertainties, or on the significant changes in child mortality and mortality among adults of working ages, associated with the impact of HIV, TB, and malaria on Millennium Development Goals (MDGs) 4 and 6. It draws upon one meta-analysis of ARVs suggesting 62% retention at 24 months, but our recent country reviews suggest most have moved between districts rather than have died, and mortality rates were often less than 10% [11]–[13]. Removing a “counterfactual” of TB deaths is unclear and difficult to explain why TB deaths in 1995 might be reduced from deaths reported now, unless we are evaluating a global TB strategy. The aim of The Global Fund's lives saved figure is not to evaluate a particular strategy, as McCoy and colleagues suggest, nor to “attribute” lives saved to the agency, but to highlight the lives saved each year from services delivered by the programs we support. The adjustment of ITNs based on data on use is important—WHO reports this as over 90% from surveys [14], and we will further adjust our estimates of lives saved as partner estimates from WHO and UNAIDS on the key parameters are updated. However, we believe McCoy and colleagues overstate the figures on ARV retention, ITN use, and issues of removing TB deaths from the 1990s.

The Global Fund is investing in improved country financial data, including funding national health accounts to improve data on spending on health, HIV, TB, and malaria by different partners in 46 countries. At present, it would not be accurate to use the reported share of total health or disease expenditure to attribute lives saved to an agency. Our communications on lives saved [3],[4] are clear that the estimates aim to show the lives saved of the programs we support together with other partners, civil society, and country HIV, TB, and malaria programs themselves. As described above and in our publications [1],[3], we use extensive criteria to exclude countries where The Global Fund does not provide significant financial and programmatic support. We stress that we play an important financing role, but the results are first and foremost those of country HIV, TB, and malaria programs. We will communicate this more clearly going forward and as we refine our methods with improved country data.

Finally, we understand the argument on vertical programs by McCoy and colleagues that reporting on individual HIV, TB, and malaria services can distort health priorities. The Global Fund is clear that it encourages countries to align funding with their health and disease strategies, and uses indicators of individual HIV, TB, and malaria services to measure progress and performance. However, we do think clear targets on HIV, TB, and malaria are important, as shown by the MDGs.

Lives saved is an important measure for health programs. We have based our estimates on real, individual verified data on a limited set of services, which have clear, documented mortality outcomes. We welcome the paper by McCoy and colleagues in this week's *PLOS Medicine*, as it discusses more fully the assumptions, explores the potential pitfalls in communication, and stresses the importance of investments in country financial and impact data. Our new evaluation plan fully supports this country investment in country data and analysis. We will update our global estimates with country data from our impact studies in 25 countries where 65% of the global burden of HIV, TB, and malaria occurs. Through these investments in global estimates and country measurement, we are confident the programs The Global Fund supports will save more than the 8.7 million lives estimated so far.

## Abbreviations

ARV: antiretroviral
DOTS: directly observed TB treatment
ITN: insecticide-treated bednet
MDG: Millennium Development Goal
